# Going beyond the Control of Quorum-Sensing to Combat Biofilm Infections

**DOI:** 10.3390/antibiotics5010003

**Published:** 2016-01-09

**Authors:** Wolf-Rainer Abraham

**Affiliations:** Chemical Microbiology, Helmholtz Center for Infection Research, Inhoffenstrasse 7, Braunschweig 38124, Germany; wolf-rainer.abraham@helmholtz-hzi.de; Tel.: +49-531-6181-4300; Fax: +49-531-6181-4699

**Keywords:** biofilm infection, quorum sensing, biofilm dispersal

## Abstract

Most bacteria attach to surfaces where they form a biofilm, cells embedded in a complex matrix of polymers. Cells in biofilms are much better protected against noxious agents than free-living cells. As a consequence it is very difficult to control pathogens with antibiotics in biofilm infections and novel targets are urgently needed. One approach aims at the communication between cells to form and to maintain a biofilm, a process called quorum-sensing. Water soluble small-sized molecules mediate this process and a number of antagonists of these compounds have been found. In this review natural compounds and synthetic drugs which do not interfere with the classical quorum-sensing compounds are discussed. For some of these compounds the targets are still not known, but others interfere with the formation of exopolysaccharides, virulence factors, or cell wall synthesis or they start an internal program of biofilm dispersal. Some of their targets are more conserved among pathogens than the receptors for quorum sensing autoinducers mediating quorum-sensing, enabling a broader application of the drug. The broad spectrum of mechanisms, the diversity of bioactive compounds, their activity against several targets, and the conservation of some targets among bacterial pathogens are promising aspects for several clinical applications of this type of biofilm-controlling compound in the future.

## 1. Pathogens in Biofilms Are Well Protected against Antibiotics and the Immune System

Most bacteria attach to surfaces and interfaces where they form a biofilm [1]. Here they are embedded in a complex matrix of polymeric substances which allow the formation of micro-niches and the maintenance of steep chemical gradients. Biofilm formation is a major problem in human health and all implants but also mucosa, e.g., in the cystic fibrosis lung, are prone to colonization by pathogens [2]. If pathogens are present in such biofilms we are dealing with an infection which is difficult to cure. Often antibiotics, even in high doses, do not eradicate the infection and for far too many implants it means replacement of the implant with the hope that the new one will not become infected again. Biofilm infections cause prolonged suffering of the patients and even can lead to their death. These infections also cause high health costs worldwide, e.g., in the U.S. alone $5000 and $34,000 per infection and resulting in more than $5 billion in added medical costs per annum [3].

Forming biofilms is the answer of micro-organisms to hostile environments. The optimal protection of the embedded cells against noxious agents, e.g., antibiotics and the immune system, is the main reason why biofilm infections are so difficult to treat. It has been found that for the eradication of pathogens from biofilms more than 1000 times higher antibiotic concentrations were required than for the same strain living in planktonic form in the serum [4]. The mechanisms of how the cells protect themselves against antibiotics are still not very well understood but several strategies seem to work hand in hand. Some reasons are: changes in gene expressions in biofilms compared to the planktonic cells, slower growing bacteria and with the reduced metabolic activity lower sensitivity against most antibiotics [5], degradation of antibiotics [6], and complexation of antibiotics by components of the biofilm matrix [7] or their active transport out of the cells [8].

## 2. Communication Mediated by Small Molecules Is Required for Biofilm Formation and Maintenance

**Figure 1:**
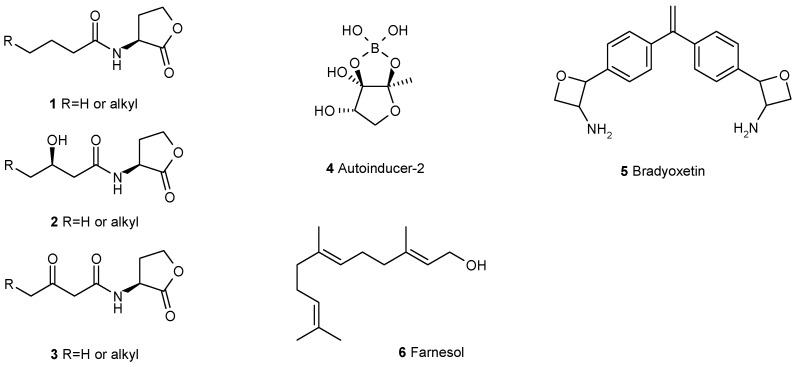
Some of the well studied autoinducers mediating quorum-sensing in bacteria and fungi.

To form a biofilm requires coordinated gene expression of the individual cells. It has been demonstrated for a still growing number of bacteria that a strategy, called quorum sensing, regulates this process [9]. The individual bacterial cell produces one or more low-molecular compounds which are transported out of the cell. At the same time the cell measures the concentration of this compound in the surrounding medium. If the cell is alone the produced compound simply diffuses into this medium and disappears. If, however, several cells produce a given compound, the concentration becomes significant. When a certain threshold of the exported compound is reached, the compound triggers in the cell a signaling cascade which leads to the induction of several genes. Because this induction in the cell is caused by compounds produced by the same cell, such compounds are also called autoinducers. We know now a number of these autoinducers and acyl-homoserine lactones (AHL) **1**–**3** are probably the best studied class of them [10]. However, in addition, several more classes of autoinducers for quorum sensing are known, they are autoinducer-2 **4**, a boron-bearing compound [11], bradyoxetin **5** [12], several diketopiperazines, e.g., **25**–**26**, farnesol **6** [13] (Figure 1), *cis*-2-alkenoic acids [14], e.g., **34**–**41**, and a variety of peptides [15], e.g., **11**–**13**, many of which are cyclic compounds [16].

These autoinducers, however, do not only coordinate individual cells to initiate biofilm formation. Autoinducers have also an important role in the maintenance of already established biofilms. In most biofilms not a single bacterial species but microbial communities of pathogens and non-pathogens grow together [17]. Here, some autoinducers also function in the communication between different species. Because of these roles autoinducers for quorum sensing are an important target for the control of biofilm infections. Numerous studies have shown that inhibiting the production of these autoinducers or blocking their receptor proteins lead to thinner and less structured biofilms which are much easier destroyed by the immune system. Hence, blocking quorum sensing became an important target for the search of biofilm modulating compounds [18]. For the majority of the studied species not only one but several autoinducers have been detected, produced in varying amounts, probably for fine-tuning of the cellular responses [19]. This, however, sets high standards for drugs to block quorum sensing effectively and reliably.

## 3. Interfering with the Communication between Microbial Cells Weakens Biofilms

### 3.1. Blocking Quorum Sensing

The communication of the individual cells is essential for the formation of biofilms, therefore, blocking this process is an important goal for the control of biofilm infections. Here, first a very brief overview on quorum sensing inhibition is given and then the focus is on compounds modulating biofilms beyond a mere quorum sensing interference. Probably because quorum sensing based on acyl-homoserine lactones is still the most studied quorum sensing system a large number of compounds, antagonistic to acyl-homoserine lactones, have been reported [20,21], e.g., the synthetic furanone **7** derived from natural compounds produced by the red macroalga *Delisea pulchra* [22]. Several of them were derived from acyl-homoserine lactones but even more were found in large chemical libraries and subsequent optimization of the hits obtained from their high-throughput screening [23]. Interesting is that a number of well known natural compounds from food, e.g., eugenol **8** [24], curcumin **9** [25], and ajoene **10** from garlic [26], can also block receptors of acyl-homoserine lactones. It is tempting to speculate that preferences in food may also be a factor for the susceptibility of individuals to biofilm infections leading directly into the field of functional food.

**Figure 2:**
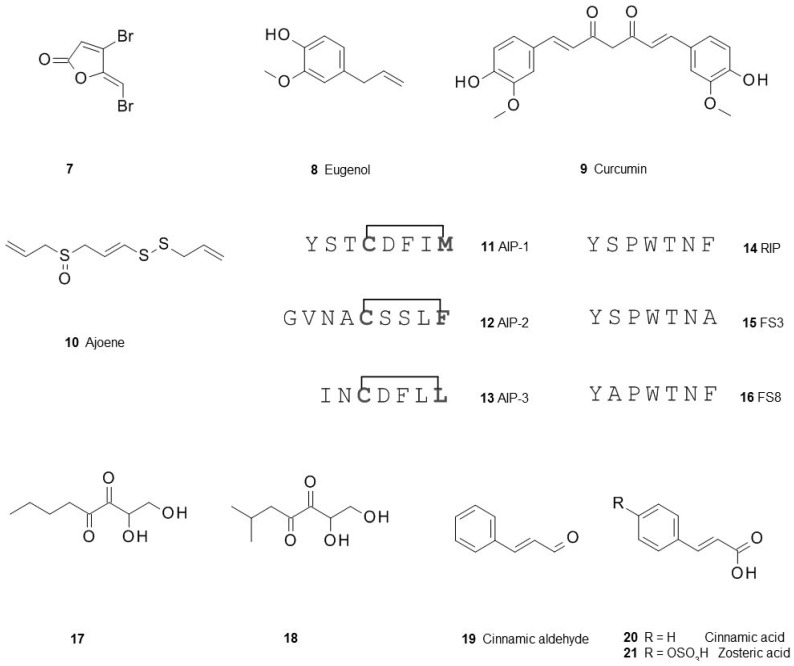
Antagonist of autoinducers of quorum-sensing competing with acyl-homoserine lactones, autoinducer-2 or autoinducer peptides (AIP) of *Staphylococcus aureus*.

*Staphylococcus* species form biofilms and are important pathogens in the clinic. Finding antagonists for their cyclic peptides mediating biofilm formation is therefore an attractive goal [27]. One of these compounds is the peptide RIP **14** [28], others, e.g., FS3 **15** [29] or FS8 **16** (Figure 2), were found after further optimization [30].

Contrary to acyl-homoserine lactones autoinducer-2 mediates quorum sensing not only within the same species but also between different, phylogenetically only distantly related species. Intervention in this communication could give a handle to control biofilm communities as has recently been demonstrated for the gut microbiota in an animal model [31]. When a series of analogs were tested for their inhibition of the autoinducer-2 signaling pathway only butyl- and isobutyl-4,5-dihydroxy-2,3-pentanedione **17** and **18** showed this inhibition both in *Escherichia coli* and in *Salmonella typhimurium* [32]. A similar effect was also found for cinnamic aldehyde **19** [33,34]. Cinnamic aldehyde is natural product found in many food products and its activity underlines again the notion that several organisms produce compounds which may help them to control biofilms.

**Figure 3:**
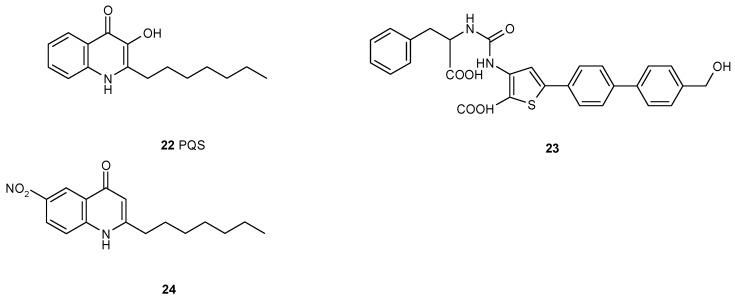
Structures of the *Pseudomonas* quinolone signal (PQS) **22** and two of its recently found inhibitors.

*Pseudomonas aeruginosa* is a biofilm forming pathogen which causes many infections and it is difficult to control because of antibiotic resistance when organized in biofilms. *P. aeruginosa* utilizes several quorum sensing systems, one of them is mediated by the unique and species-specific *Pseudomonas* Quinolone Signal PQS **22**. Not many compounds antagonizing PQS have been described, some of them are 2-heptyl-4-hydroxy-6-nitro-quinoline **24** [35] or the ureidothiophene-2-carboxylic acid **23** [36] (Figure 3).

There are several reports which question the effect of reported quorum quenching compounds. When assessing the results of quorum quenching studies one should be aware that the majority of these studies have been done using reporter strains. The reported results can only be compared if standardized control experiments have been done and the toxicity of the tested compounds on the reporter organism has been determined. For a number of reports it is not completely clear whether this is an effect caused by inhibition of quorum sensing or simply by the antibiotic effect of the compounds under study [37].

### 3.2. Multiple Interferences of Quorum Sensing Inhibitors with Biofilm Formation

Using reporter strains for the detection of quorum sensing inhibition allows high-throughput screening of large compound libraries. Quorum sensing, however, is only one of the characteristics of biofilms and the aim is not the interruption of cell-cell communication *per se* but the prevention of biofilm formation or the dispersal of already established biofilms. The latter is usually the aim in medicine where patients have already developed well established biofilms before showing any clinical symptoms. Therefore, compounds are discussed here in more detail showing effects beyond inhibition of the various quorum sensing cascades.

**Figure 4:**
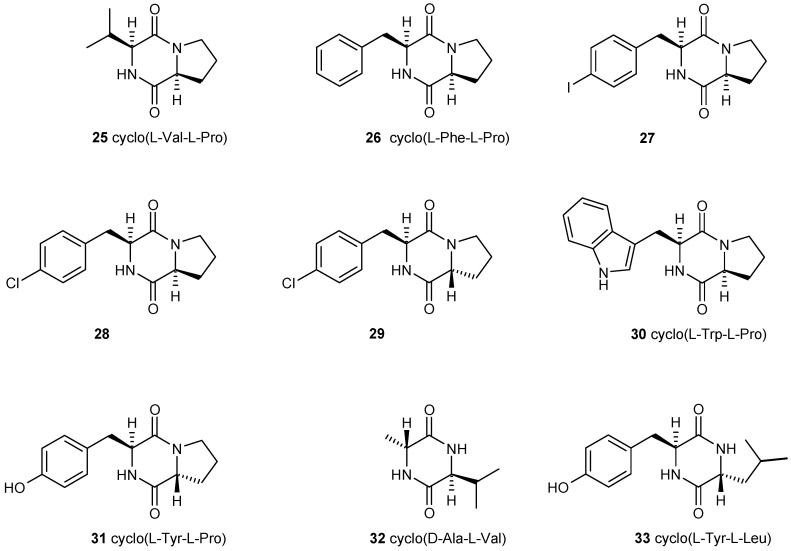
A number of bioactive cyclic dipeptides have been described; some of them also seem to have a role in quorum-sensing. From these compounds inhibitors have been developed. Many of these compounds mediate interspecies interactions.

It has been reported that a number of cyclic dipeptides, produced by many organisms [38], have an effect on biofilm formation. Holden *et al.*, demonstrated that cyclo(l-Val-l-Pro) **25** can activate the homoserine lactone biosensor although considerable higher concentrations are needed than for the natural homoserine lactone [39]. Campbell synthesized several cyclic dipeptides and tested this library against a Vibrio fischeri reporter strain but could not confirm these results. However, the synthetic cyclo(l-4-iodo-Phe-l-Pro) **27** and cyclo(l-4-chloro-Phe-l-Pro) **28** were inhibitors of quorum sensing mediated luminescence and cyclo(l-4-chloro-Phe-d-Pro) **29** and cyclo(l-Trp-l-Pro) **30** were moderate inhibitors [40]. For other cyclic dipeptides also quorum quenching both in Gram-positive and -negative bacteria has been reported. Cyclo(l-Tyr-l-Pro) **31** and cyclo(d-Ala-l-Val) **32** reduced colony expansion in a strain of *Serratia liquefaciens* [41]. *Lactobacillus reuteri* produces cyclo(l-Phe-l-Pro) **25** and cyclo(l-Tyr-l-Pro) **31** which strongly inhibit quorum sensing in *Staphylococcus aureus* [42] and from a marine *Penicillium* sp. cyclo(l-Tyr-l-Leu) **33** (Figure 4) has been isolated inhibiting biofilm formation of *Staphylococcus epidermidis* [43]. These somewhat contradicting results shed some doubts on the role of cyclic dipeptides as quorum sensing mediators. However, they also make it very likely that cyclic dipeptides act in interspecies communications because these compounds have been detected in many organisms.

Looking only for inhibition of receptors of the autoinducers may be misleading because some compounds have more than one target in the biofilm. As already mentioned, curcumin **9** inhibits quorum sensing by blocking AHL-sensors [44] but this is not the only effect curcumin has on cells organized in biofilms. This natural product also inhibits sortase A in *Streptococcus mutans*, a membrane-localized transpeptidase possessing an important role in adherence that has been associated with cariogenicity [45,46]. It remains to be determined whether the consumption of curry or food colored with curcumin, has an influence on biofilm formation and on oral health of the consumers.

### 3.3. Dispersal of Biofilms

To form a biofilm gives the bacteria protection and allows the production of virulence factors. However, a biofilm can also be a trap for the inhabitants when conditions become unfavorable. To overcome this problem most microbes developed strategies to dissolve their biofilms and allow the embedded cells to move to more convenient sites. Regarding the fact that biofilms have already been established when clinical symptoms are shown, using these biofilm dissolving strategies would be of high priority to combat biofilm infections. Nitric oxide is produced by nitrite reductases in the biofilm to induce dispersal [47,48]. This has been observed for a wide range of pathogens, e.g., *Pseudomonas aeruginosa*, *Escherichia coli*, *Vibrio cholera*, *Neisseria gonorrhoeae* [49], and *Staphylococcus aureus* [50] but also in multispecies biofilms [51] and even in fungi [52]. These findings indicate that this mode of dispersion is rather widespread. Although it is tempting to use NO for the control of biofilm infections one has to be aware that increased NO concentrations have also a number of side effects, e.g., immunosuppression, inhibition of angiogenesis [53] or even cytotoxicity via nitrosylation of proteins [54]. This has been confirmed in a clinical study involving treatment of patients with cystic fibrosis with NO gas [55]. One way to overcome these problems is the delivery of NO on site without flushing the whole organism with this reactive compound. For this purpose diazeniumdiolates have been developed which can be used as a pro-drug or embedded in polymers for the protection of implants [56]. A very sophisticated variation of this approach is the coupling of diazeniumdiolates to cephalosporin from where it is released by bacterial β-lactamases [57]. For the local protection of implants from colonization by biofilms, several NO-releasing polymers have been designed, e.g., dendrimers [58] or sol-gels [59]. However, when considering the application of these materials in implants one should keep in mind that the release of NO should dissolve evolving biofilms but the concentration should be kept low enough not to hinder the regeneration of host cells. The field of NO-application in pro-drugs is wide and still under development [60].

**Figure 5:**
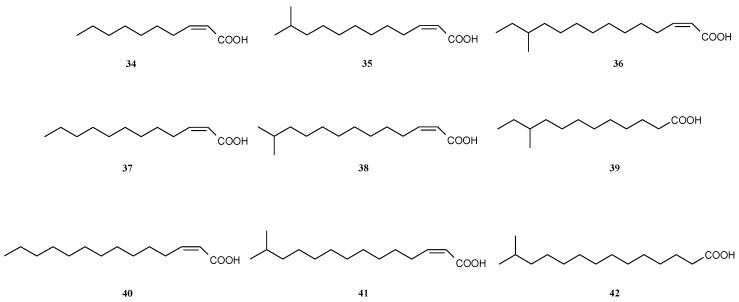
Many *cis*-2-alkenoyl acids mediate the communication between different species but *cis*-2-decenoic acid has been found to trigger the process of biofilm dispersal in several species including *Pseudomonas aeruginosa*.

When exposed to spent medium, biofilms of *Pseudomonas aeruginosa* dispersed. The compound triggering this reaction was identified as *cis*-2-decenoic acid **34** [61] (Figure 5). Similar *cis*-2-alkenoic acids have been isolated from *Xanthomonas campestris, Stenotrophomonas maltophilia,* and *Burkholderia* species and this class of compounds is termed diffusible signaling factors (DSF). In general, considerably higher activities have been observed for the *cis*-2-alkenoic acids compared to their saturated analogues.DSFs have a number of effects in the cells but they also mediate interspecies interactions. *cis*-2-Dodecenoic acid **37** increases in *Burkholderia cenocepacia* motility and biofilm formation but also virulence [62]. Interestingly, the AHL-quorum sensing system and that of DSF seem to interact [63]. Comparative transcriptome analysis in *Pseudomonas aeruginosa* demonstrated a large number of genes being differently regulated by *cis*-2-decenoic acid **34** [64], however, a clear pattern explaining the altered phenotype could not be deduced from these experiments. From the opportunistic pathogen *Stenotrophomonas maltophila* several fatty acids (**35**–**36**, **38**–**41**) have been identified which facilitate movement of its cells [65] and mediate the communication between *Stenotrophomonas maltophila* and the pathogen *Pseudomonas aeruginosa*. The fatty acids produced by *S. maltophila* increase the tolerance to cationic antimicrobial peptides in *P. aeruginosa* [66]. Interestingly, 12-methyl-tetradecanoic acid **42**, not found in *Stenotrophomonas maltophila,* blocked swarming motility completely at 10 µg·mL^−1^ in *Pseudomonas aeruginosa* and led to reduced biofilm formation by 31% [67]. Another DSF, *cis*-2-dodecenoic acid **37**, enabled bacteria-fungus interaction as was shown between *Burkholderia cenocepacia* and *Candida albicans* [68]. The same fatty acid down-regulates biofilm formation in *Pseudomonas aeruginosa* and inhibits its type-III secretion system [69].

**Figure 6:**
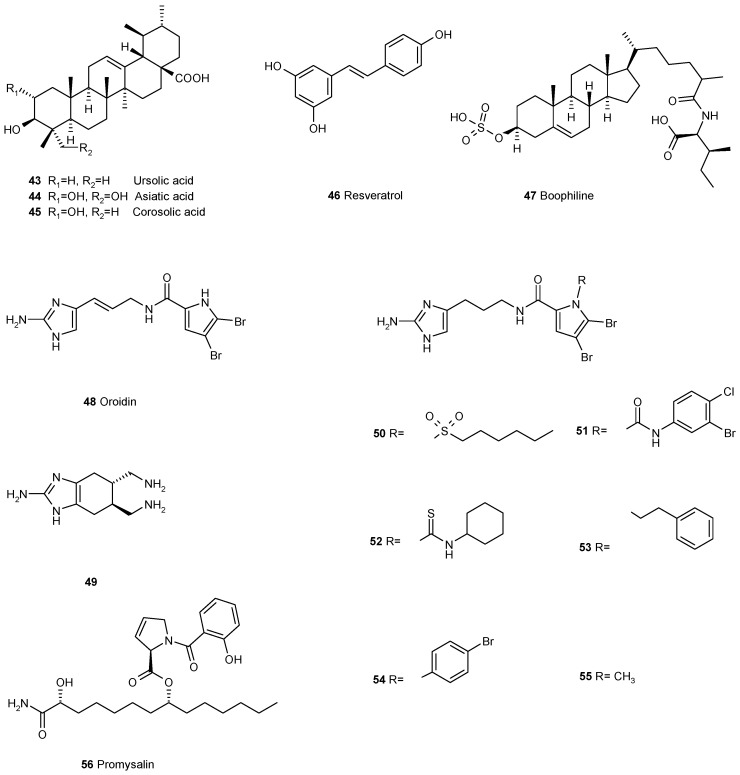
A number of natural products and their derivatives have been found to prevent the formation of biofilms or to disperse established ones but their mechanisms of action are still unknown.

The biosyntheses of the DSFs is not yet fully understood. In *Pseudomonas aeruginosa* the involvement of the putative enoyl-coenzyme A hydratase DspI, showing significant homologies to the protein RpfF of *Xanthomonas campestris,* has been identified [70]. *cis*-2-Dodecenoic acid **37** of *Burkholderia cenocepacia* is synthesized from the acyl carrier protein (ACP) thioester of 3-hydroxydodecanoic acid which is then converted by the enzyme RpfF first to *cis*-2-dodecenoyl-ACP. However, RpfF catalyzes not only dehydration of 3-hydroxydodecanoyl-ACP to *cis*-2-dodecenoyl-ACP but also cleaves the thioester to release the free acid [71].

The best understanding of the mechanism of action has been achieved for *X. campestrensis*. Here, synthesis and perception of DSF require products of the *rpf* gene cluster (for regulation of pathogenicity factors). Similar gene clusters have also been found in many but not all DSFs producing bacteria. The sensor kinase RpfC of *Xanthomonas campestris,* anchored in the cytoplasmic membrane, consists of a membrane spanning sensory domain, a histidine kinase domain, an ATP kinase binding domain, followed by a receiver domain, and a histidine phosphotransfer domain. After binding of DSF the histidine kinase domain is autophosphorylated requiring ATP. The phosphorelay signalling then leads to the phosphorylation of the cyclic di-GMP phosphodiesterase RpfG causing changes in the cyclic di-GMP level in the cell which in turn affects, e.g., the synthesis of virulence factors or causes biofilm dispersal [72]. The sensor kinase RpfR of *Burkholderia cenocepacia* becomes a potent c-di-GMP phosphodiesterase leading to the control of several but not all DSF-influenced genes [73]. Analysis of 82 *Stenotrophomonas maltophilia* clinical isolates, revealed the presence of two distinct classes of *rpfC*-*rpfF* genes. Only one class was able to form DSF while the other one seemed to permanently repress DSF-formation demonstrating that DSF-signaling is not essential for infection [74].

Because of their ability to disperse existing biofilms DSFs are receiving growing attention. They have also been reported to increase metabolic activity and restore antimicrobial susceptibility of persister cells [75], both important conditions for the control of biofilm infections. In another study, the inhibition of rhamnolipid synthesis and biofilm formation in *Pseudomonas aeruginosa* by 2-bromoalkanoic acids was evaluated. Here stronger effects of 2-bromohexanoic acid compared to 2-bromooctanoic acid were observed, while 2-bromodecanoic acid showed the weakest effect [76]. It should be generally stated here that for rigorous assessment of results we need standardized protocols to avoid contradicting findings, e.g., Davies *et al.*, reported dispersion of *S. aureus* biofilms with 10 nM of *cis*-2-decenoic acid but Su *et al.*, needed about 200 µM to achieve this effect [77].

### 3.4. Other Ways of Biofilm Prevention or Dispersal

The triterpene ursolic acid **43** (Figure 6) inhibits biofilm formation at 10 μg·ml^-1^ in *Escherichia coli*, *Pseudomonas aeruginosa* and *Vibrio harveyi* but it does not act via quorum sensing, as has been demonstrated with the *V. harveyi* reporter system [78]. Ursolic acid also inhibited methicillin-resistant *Staphylococcus aureus* (MRSA) biofilm formation but had no effect on established biofilms, whereas resveratrol **46** inhibited MRSA biofilm formation and could partially remove established biofilms. RNA-Seq-based transcriptome analysis of MRSA biofilm inhibition by ursolic acid and resveratrol revealed two different mechanisms. While ursolic acid inhibited biofilm formation by reducing amino acids metabolism and adhesin expressions resveratrol disturbed quorum sensing and the synthesis of surface proteins and capsular polysaccharides [79]. The closely related asiatic acid **44** and corosolic acid **45** are even more effective and they also increase the susceptibility of *Pseudomonas aeruginosa* to antibiotics [80].

Following the hypothesis that the majority of all higher organisms have developed strategies to protect themselves against biofilm infections the protection mechanisms of ticks (*Rhipicephalus microplus*) were investigated. It was observed that their eggs were not infected by *Pseudomonas aeruginosa* and from tick eggs’ extract the cholesterol derivative boophiline **47** was isolated. Boophiline was found to be a weak antibiotic but it had not yet been connected to biofilms [81]. A detailed study on the effect of boophiline on *Pseudomonas aeruginosa* revealed that it did not interfere with the quorum sensing circuits of the pathogens. Instead, strong inhibitions of the expression of *cdrA* (an adhesin, required as biofilm scaffold) and *fliC* (flagellin, required as flagellar filament) were found. The bactericidal effect was observed for *Staphylococcus epidermidis* and related Gram-positive bacteria [82]. These observations point to different targets of the molecule enabling the inhibition of biofilm formation by a broad variety of bacteria.

The alkaloid oroidin **48**, discovered in the sponge *Agelas oroides* [83], was shown to modulate biofilm formation of a number of bacteria [84]. There seems to be no report on the mode of action of this compound to biofilms. Taking oroidin as a lead, several bioactive compounds have been synthesized and evaluated for their inhibition of biofilm formation [85]. The sulphonamide **50**, the urea **51,** and the thiourea **52** analogues were demonstrated to inhibit selectively *P. aeruginosa* biofilm formation, while being nontoxic to *Caenorhabditis elegans* [86]. The derivatives **53** and **54** were both active against biofilm formation of the pathogens *Acinetobacter baumannii*, *Pseudomonas aeruginosa* PA14, and *Bordetella bronchiseptica* [87]. Inhibition by oroidin derivatives was observed not only for Gram-negative but also for Gram-positive bacteria biofilm. The conformational constrained indole-based analogue of oridin **49** was active against methicillin-resistant *Staphylococcus aureus* and *Streptococcus mutans* and suppressed biofilm formation in the lower micromolar range [88]. One of the most active compounds was the *N*-methylated derivative **55** of dihydro-oridin. Maybe some light can be shed on the mode of action of these compounds by their activity against *Porphyromonas gingivalis*. *P. gingivalis*, one of the central organisms in periodontal disease, which binds to the primary colonizer *Streptococcus gordonii*. Three small molecules derived from oroidin and containing 2-aminoimidazole or 2-aminobenzimidazole moieties inhibited binding of *P. gingivalis* to *S. gordonii* by reducing expression of fimbrial adhesins, necessary for *P. gingivalis* adhesion to *S. gordonii* [89].

Zosteric acid **21** has been reported to reduce biofilm formation of the bacteria *Escherichia coli, Bacillus cereus,* and *Pseudomonas aeruginosa* [90] and the fungi *Aspergillus niger, Penicillium citrinum*, and *Candida albicans* [91]. An increased flagellin amount in *E. coli* leading to increased formation of flagella has been identified as one of the mechanisms. This is probably the reaction of the cells to a stress condition caused by zosteric acid [92]. Screening of a small library of zosteric derivatives showed that cinnamic acid **20**, lacking the *p*-sulfoxy-moiety, was even more active. Using an *E. coli* protein pull-down approach the enzyme NADH: quinone dehydrogenase was identified as the target molecule for these compounds. This is a tetrameric flavoprotein with a hydrophobic active site pocket which is most likely the interaction site for these cinnamic acids [93].

*Pseudomonas putida* produces promysalin **56** which acts only against some *Pseudomonas* species, among them the pathogen *P. aeruginosa* [94]. A recent study demonstrated that promysalin acts as a biosurfactant on established biofilms but it also suppresses the production of the virulence factor pyoverdine [95]. The mere action as a biosurfactant is probably not sufficient to explain the remarkable species-selectivity of the compound and the underlying mechanism remains to be discovered.

All higher organisms have to deal with biofilm infections and they have developed a multitude of mechanisms to control biofilm communities. These examples of natural compounds acting beyond the simple competition for the receptors of the known quorum sensing autoinducers demonstrate that there is a vast and still poorly explored field of targets for biofilm control. However, the examples show that several of these compounds have more than one target and the transition between biofilm control *vs*. antibiotic *vs*. pathoblocker [96] seems to be smooth. To find these compounds one has to leave screening with genetic constructs which focus only on the inhibition of acyl-homoserine lactone- or AI-2 receptors and one has to go instead to less specific screens.

### 3.5. Combinations with Antibiotics

Dissolving an existing biofilm may be sufficient to allow clearance of its inhabiting pathogens by the immune system. However, for an inefficient or overloaded immune system of a patient the release of pathogens from biofilms may even have a detrimental effect turning an infection from a chronic to an acute state and fostering the production of virulence factors [97]. Because most biofilm-dissolving drugs do not kill the bacteria their combination with antibiotics is a logical consequence. Rasmussen *et al.*, tested patulin in *Pseudomonas aeruginosa* as an antagonist of acylhomoserine lactone. While patulin had no effect on the survival of *P. aeruginosa* cells in the bofilm and treatment of the biofilm with the antibiotic tobramycin could kill only few cells, the combination of patulin and tobramycin led to severe killing of the cells [98]. The RNA-II inhibiting peptide FS 8 **16** reduced the formation of biofilms and virulence factors of *Staphylococcus aureus* but did not kill the pathogen. The combination of this quorum-quenching compound with the antibiotic tigecycline in a rat model, however, increased the sensitivity of *S. aureus* four-fold compared to tigecycline alone [30].

Biofilms of *Pseudomonas auroginosa* can be dissolved by *cis*-2-decenoic acid **34** but the bacteria are not killed. When pre-established biofilms of St*aphylococcus aureus*, *Bacillus cereus*, *Salmonella enterica* or *Escherichia coli* were treated with desinfectants or antibiotics only moderate reductions in living cells were achieved. The addition of only 310 nM *cis*-2-decenoic acid to these toxins caused a significant increase of cell eradication, e.g., for *S. aureus* and ciprofloxacin it increased from 11% for the antibiotic alone to 87% for the combination [99]. Similar observations have been made for other bacteria, e.g., *Mycobacterium smegmatis*, *Neisseria subflava* or *Bacillus thuringiensis* [100] or in mixed biofilms containing the pathogen *Klebsiella pneumonia* [101]. Against methicillin-resistant *Staphylococcus aureus* or *Acinetobacter baumannii* 4,5-disubstituted-2-aminoimidazole-triazole conjugates were developed which inhibited their biofilm formation. When combined with antibiotics they also re-sensitized the pathogens for eradication with these antibiotics [77].

## 4. The Long Way to Go

Often it has been argued that biofilm-interfering compounds will not lead to the development of resistances in the pathogens because preventing or dispersing biofilms is not lethal for the microbes [102]. Although such a notion is probably correct in some aspects it should be kept in mind that formation of biofilms is a protection mechanism against a hostile environment. Microorganisms which are capable in forming biofilms have an advantage against those living always in the planktonic form. This will cause some selective pressure towards biofilm-formers although the pressure will be probably less than for the evasion from killing by antibiotics [103].

Currently, the often high specificity of quorum quenching drugs limits their application [104], especially against microbial communities. Sometimes even closely related species do not respond to these drugs, dramatically increasing the chances for failure of such a treatment. The consequence is that compounds with such limited spectra against pathogens may not find their way into the market. Beside a sufficiently broad specificity of the drug, its stability within the biofilm matrix will become an issue. From many studies it is known that gene expression is largely altered in biofilms and this also includes enzymes which may act as potential degraders of the drug in question [105].

For the application of an anti-biofilm compound it is not only important that it is active against the pathogen but also that it does not do any damage to the host. Sadly, for some of the reported compounds this aspect has been ignored but for the evaluation of a novel drug the assessment of its cytotoxicity is mandatory [106]. Biocompatibility is an issue for drugs applied for the prevention of biofilms on implants. This can be done by testing their effects on dedicated cells [107] or by applying them in animal experiments. Local drug release [108] may be an attractive option especially for biofilm-preventing compounds on implants. It concentrates the drug to the site of the implantation but it requires the control of the release in order to avoid toxic concentrations. When Rawson *et al.*, tested biofilm modulating compounds for their influence on osteoblast differentiation they found *cis*-2-decenoic acid **34** biocompatible but farnesol **6** induced cytotoxic responses within their biofilm inhibitory concentrations [109]. Many different approaches are nowadays available to achieve controlled drug release.

## 5. Conclusions

Biofilms have complex and dynamic structures mostly composed of several microbial species. The complexity of biofilms helps them to survive under harsh conditions but it also offers a multitude of approaches for their control. To prevent or to cure biofilm infections the main focus has been set to quorum-sensing. Screening for quorum-quenching compounds can nowadays be done in high-throughput screenings because several assays targeting the formation of autoinducers or their receptors are available. However, the entire mechanism for quorum-sensing is known only for a small number of pathogens and we still know very little about interspecies cross-talks. This article had the intention to show that many organisms have developed compounds which interfere with biofilms and many do this outside the known quorum-sensing circuits. Because these natural compounds have a variety of targets or their targets are even not yet known, screening for these bioactive compounds is more laborious and slower. The reward for such an effort, however, is the discovery of new targets and new mechanisms as have been highlighted for *cis*-2-decenoic acid **34**, ursolic acid **43** or boophiline **47**, only to mention a few. Some of the compounds so discovered have several targets allowing the control of a broader spectrum of pathogens. It is certainly not a risk to predict that in the coming years, novel mechanisms of biofilm dispersal or blocking of antibiotic resistance in biofilms will be uncovered. Combining these biofilm controlling compounds with established or novel antibiotics will add another dimension to the treatment of biofilm infections.

## References

[1] Costerton J.W., Cheng K.J., Geesey G.G., Ladd T.I., Nickel J.C., Dasgupta M., Marrie T.J. (1987). Bacterial biofilms in nature and disease. Annu. Rev. Microbiol..

[2] Singh P.K., Schaefer A.L., Parsek M.R., Moninger T.O., Welsh M.J., Greenberg E.P. (2000). Quorum-sensing signals indicate that cystic fibrosis lungs are infected with bacterial biofilms. Nature.

[3] Wenzel R.P. (2007). Health care-associated infections: Major issues in the early years of the 21st century. Clin. Infect. Dis..

[4] Olsen I. (2015). Biofilm-specific antibiotic tolerance and resistance. Eur. J. Clin. Microbiol. Infect. Dis..

[5] Jolivet-Gougeon A., Bonnaure-Mallet M. (2014). Biofilms as a mechanism of bacterial resistance. Drug Discov. Today Technol..

[6] Cantón R., Morosini M.I. (2011). Emergence and spread of antibiotic resistance following exposure to antibiotics. FEMS Microbiol. Rev..

[7] Mah T.-F., Pitts B., Pellock B., Walker G.C., Stewart P.S., O’Toole G.A. (2003). A genetic basis for *Pseudomonas aeruginosa* biofilm antibiotic resistance. Nature.

[8] Sun J., Deng Z., Yan A. (2014). Bacterial multidrug efflux pumps: Mechanisms, physiology and pharmacological exploitations. Biochem. Biophys. Res. Commun..

[9] Waters C.M., Bassler B.L. (2005). Quorum sensing: Cell-to-cell communication in bacteria. Annu. Rev. Cell Dev. Biol..

[10] Wang Y., Ma S. (2014). Small molecules modulating AHL-based quorum sensing to attenuate bacteria virulence and biofilms as promising antimicrobial drugs. Curr. Med. Chem..

[11] Pereira C.S., Thompson J.A., Xavier K.B. (2013). AI-2-mediated signalling in bacteria. FEMS Microbiol. Rev..

[12] Loh J., Carlson R.W., York W.S., Stacey G. (2002). Bradyoxetin, a unique chemical signal involved in symbiotic gene regulation. Proc. Natl. Acad. Sci. USA.

[13] Albuquerque P., Casadevall A. (2012). Quorum sensing in fungi—A review. Med. Mycol..

[14] Deng Y., Wu J., Tao F., Zhang L.-H. (2011). Listening to a new language: DSF-based quorum sensing in Gram-negative bacteria. Chem. Rev..

[15] Monnet V., Juillard V., Gardan R. (2014). Peptide conversations in Gram-positive bacteria. Crit. Rev. Microbiol..

[16] Novick R.P., Geisinger E. (2008). Quorum sensing in staphylococci. Annu. Rev. Genet..

[17] Pichlmaier M., Marwitz V., Kühn C., Niehaus M., Klein G., Bara C., Haverich A., Abraham W.-R. (2008). High prevalence of asymptomatic bacterial colonization of rhythm management devices. Europace.

[18] Scutera S., Zucca M., Savoia D. (2014). Novel approaches for the design and discovery of quorum-sensing inhibitors. Expert Opin. Drug Discov..

[19] Jimenez P.N., Koch G., Thompson J.A., Xavier K.B., Cool R.H., Quax W.J. (2012). The multiple signaling systems regulating virulence in *Pseudomonas aeruginosa*. Microbiol. Mol. Biol. Rev..

[20] Hirakawa H., Tomita H. (2013). Interference of bacterial cell-to-cell communication: A new concept of antimicrobial chemotherapy breaks antibiotic resistance. Front. Microbiol..

[21] Jiang T., Li M. (2013). Quorum sensing inhibitors: A patent review. Expert Opin. Ther. Pat..

[22] De Nys R., Wright A.D., König G.M., Sticher O. (1993). New halogenated furanones from the marine red alga *Delisea pulchra* (*cf.* fimbriata). Tetrahedron.

[23] Estrela A.B., Heck M.G., Abraham W.-R. (2009). Novel approaches to control biofilm infections. Curr. Med. Chem..

[24] Zhou L., Zheng H., Tang Y., Yu W., Gong Q. (2013). Eugenol inhibits quorum sensing at sub-inhibitory concentrations. Biotechnol. Lett..

[25] Rudrappa T., Bais H.P. (2008). Curcumin, a known phenolic from *Curcuma longa*, attenuates the virulence of *Pseudomonas aeruginosa* PAO1 in whole plant and animal pathogenicity models. J. Agric. Food Chem..

[26] Jakobsen T.H., van Gennip M., Phipps R.K., Shanmugham M.S., Christensen L.D., Alhede M., Skindersoe M.E., Rasmussen T.B., Friedrich K., Uthe F. (2012). Ajoene, a sulfur-rich molecule from garlic, inhibits genes controlled by quorum sensing. Antimicrob. Agents Chemother..

[27] Gray B., Hall P., Gresham H. (2013). Targeting agr- and agr-Like quorum sensing systems for development of common therapeutics to treat multiple gram-positive bacterial infections. Sensors.

[28] Balaban N., Cirioni O., Giacometti A., Ghiselli R., Braunstein J.B., Silvestri C., Mocchegiani F., Saba V., Scalise G. (2007). Treatment of *Staphylococcus aureus* biofilm infection by the quorum-sensing inhibitor RIP. Antimicrob. Agents Chemother..

[29] Cirioni O., Mocchegiani F., Cacciatore I., Vecchiet J., Silvestri C., Baldassarre L., Ucciferri C., Orsetti E., Castelli P., Provinciali M. (2013). Quorum sensing inhibitor FS3-coated vascular graft enhances daptomycin efficacy in a rat model of staphylococcal infection. Peptides.

[30] Simonetti O., Cirioni O., Mocchegiani F., Cacciatore I., Silvestri C., Baldassarre L., Orlando F., Castelli P., Provinciali M., Vivarelli M. (2013). The efficacy of the quorum sensing inhibitor FS8 and tigecycline in preventing prosthesis biofilm in an animal model of staphylococcal infection. Int. J. Mol. Sci..

[31] Thompson J.A., Oliveira R.A., Djukovic A., Ubeda C., Xavier K.B. (2015). Manipulation of the quorum sensing signal AI-2 affects the antibiotic-treated gut microbiota. Cell Rep..

[32] Roy V., Smith J.A.I., Wang J., Stewart J.E., Bentley W.E., Sintim H.O. (2010). Synthetic analogs tailor native AI-2 signaling across bacterial species. J. Am. Chem. Soc..

[33] Brackman G., Celen S., Hillaert U., van Calenbergh S., Cos P., Maes L., Nelis H.J., Coenye T. (2011). Structure-activity relationship of cinnamaldehyde analogs as inhibitors of AI-2 based quorum sensing and their effect on virulence of *Vibrio* spp.. PLoS ONE.

[34] Brackman G., Defoirdt T., Miyamoto C., Bossier P., Calenbergh S.V., Nelis H., Coenyie T. (2008). Cinnamaldehyde and cinnamaldehyde derivatives reduce virulence in *Vibrio* spp. by decreasing the DNA-binding activity of the quorum sensing response regulator LuxR. BMC Microbiol..

[35] Lu C., Kirsch B., Zimmer C., de Jong J.C., Henn C., Maurer C.K., Müsken M., Häussler S., Steinbach A., Hartmann R.W. (2012). Discovery of antagonists of PqsR, a key player in 2-alkyl-4-quinolone-dependent quorum sensing in *Pseudomonas aeruginosa*. Chem. Biol..

[36] Sahner J.H., Empting M., Kamal A., Weidel E., Groh M., Börger C., Hartmann R.W. (2015). Exploring the chemical space of ureidothiophene-2-carboxylic acids as inhibitors of the quorum sensing enzyme PqsD from *Pseudomonas aeruginosa*. Eur. J. Med. Chem..

[37] Defoirdt T., Brackman G., Coenye T. (2013). Quorum sensing inhibitors: How strong is the evidence?. Trends Microbiol..

[38] De Carvalho M.P., Abraham W.-R. (2012). Antimicrobial and biofilm inhibiting diketopiperazines. Curr. Med. Chem..

[39] Holden M.T.G., Chhabra S.R., de Nys R., Stead P., Bainton N.J., Hill P.J., Manefeld M., Kumar N., Labatte M., England D. (1999). Quorum-sensing cross talk: Isolation and chemical characterization of cyclic dipeptides from *Pseudomonas aeruginosa* and other Gram-negative bacteria. Mol. Microbiol..

[40] Campbell J., Lin Q., Geske G.D., Blackwell H.E. (2009). New and unexpected insights into the modulation of LuxR-type quorum sensing by cyclic dipeptides. ACS Chem. Biol..

[41] Ryan R.P., Dow J.M. (2008). Diffusible signals and interspecies communication in bacteria. Microbiology.

[42] Li J., Wang W., Xu S.X., Magarvey N.A., McCormick J.K. (2011). *Lactobacillus reuteri*-produced cyclic dipeptides quench agr-mediated expression of toxic shock syndrome toxin-1 in staphylococci. Proc. Natl. Acad. Sci. USA.

[43] Scopel M., Abraham W.-R., Henriques A.T., Macedo A.J. (2013). Dipeptide *cis*-cyclo(leucyl-tyrosyl) produced by sponge associated *Penicillium* sp.F37 inhibits biofilm formation of the pathogenic *Staphylococcus epidermidis*. Bioorganic Med. Chem. Lett..

[44] Brackman G., Hillaert U., Calenbergh S.V., Nelis H.J., Coenye T. (2009). Use of quorum sensing inhibitors to interfere with biofilm formation and development in *Burkholderia multivorans* and *Burkholderia cenocepacia*. Res. Microbiol..

[45] Song J., Choi B., Jin E.J., Yoon Y., Choi K.H. (2012). Curcumin suppresses *Streptococcus mutans* adherence to human tooth surfaces and extracellular matrix proteins. Eur. J. Clin. Microbiol. Infect. Dis..

[46] Hu P., Huang P., Chen M.W. (2013). Curcumin reduces *Streptococcus mutans* biofilm formation by inhibiting sortase A activity. Arch. Oral Biol..

[47] Arora D.P., Hossain S., Xu Y., Boon E.M. (2015). Nitric oxide regulation of bacterial biofilms. Biochemistry.

[48] Barraud N., Hassett D.J., Hwang S.H., Rice S.A., Kjelleberg S., Webb J.S. (2006). Involvement of nitric oxide in biofilm dispersal of *Pseudomonas aeruginosa*. J. Bacteriol..

[49] Potter A.J., Kidd S.P., Edwards J.L., Falsetta M.L., Apicella M.A., Jennings M.P., McEwan A.G. (2009). Thioredoxin reductase is essential for protection of *Neisseria gonorrhoeae* against killing by nitric oxide and for bacterial growth during interaction with cervical epithelial cells. J. Infect. Dis..

[50] Schlag S., Nerz C., Birkenstock T.A., Altenberend F., Götz F. (2007). Inhibition of staphylococcal biofilm formation by nitrite. J. Bacteriol..

[51] Barraud N., Storey M.V., Moore Z.P., Webb J.S., Rice S.A., Kjelleberg S. (2009). Nitric oxide-mediated dispersal in single- and multi-species biofilms of clinically and industrially relevant microorganisms. Microb. Biotechnol..

[52] Wilken M., Huchzermeyer B. (1999). Suppression of mycelia formation by NO produced endogenously in *Candida tropicalis*. Eur. J. Cell Biol..

[53] Babaei S., Teichert-Kuliszewska K., Monge J.C., Mohamed F., Bendeck M.P., Stewart D.J. (1998). Role of nitric oxide in the angiogenic response in vitro to basic fibroblast growth factor. Circ. Res..

[54] Ridnour L.A., Thomas D.D., Mancardi D., Espey M.G., Miranda K.M., Paolocci N., Feelisch M., Fukuto J., Wink D.A. (2004). The chemistry of nitrosative stress induced by nitric oxide and reactive nitrogen oxide species. Putting perspective on stressful biological situations. Biol. Chem..

[55] Miller C. Inhaled nitric oxide. Proceedings of the 36th European Cystic Fibrosis Conference.

[56] Keefer L.K. (2011). Fifty years of diazeniumdiolate research. From laboratory curiosity to broad-spectrum biomedical advances. ACS Chem. Biol..

[57] Barraud N., Kardak B.G., Yepuri N.R., Howlin R.P., Webb J.S., Faust S.N., Kjelleberg S., Rice S.A., Kelso M.J. (2012). Cephalosporin-3′-diazeniumdiolates: Targeted NO-donor prodrugs for dispersing bacterial biofilms. Angew. Chem. Int. Ed..

[58] Sun B., Slomberg D.L., Chudasama S.L., Lu Y., Schoenfisch M.H. (2012). Nitric oxide-releasing dendrimers as antibacterial agents. Biomacromolecules.

[59] Nablo B.J., Rothrock A.R., Schoenfisch M.H. (2005). Nitric oxide-releasing sol-gels as antibacterial coatings for orthopedic implants. Biomaterials.

[60] Barraud N., Kelso M.J., Rice S.A., Kjelleberg S. (2015). Nitric oxide: A key mediator of biofilm dispersal with applications in infectious diseases. Curr. Pharm. Des..

[61] Davies D.G., Marques C.N.H. (2009). A fatty acid messenger is responsible for inducing dispersion in microbial biofilms. J. Bacteriol..

[62] Ryan R.P., McCarthy Y., Watt S.A., Niehaus K., Dow J.M. (2009). Intraspecies signaling involving the diffusible signal factor BDSF (*cis*-2-dodecenoic acid) influences virulence in *Burkholderia cenocepacia*. J. Bacteriol..

[63] Udine C., Brackman G., Bazzini S., Buroni S., van Acker H., Pasca M.R., Riccardi G., Coenye T. (2013). Phenotypic and genotypic characterisation of *Burkholderia cenocepacia* J2315 mutants affected in homoserine lactone and diffusible signal factor-based quorum sensing systems suggests interplay between both types of systems. PLoS ONE.

[64] Rahmani-Badi A., Sepehr S., Fallahi H., Heidari-Keshel S. (2015). Dissection of the *cis*-2-decenoic acid signaling network in *Pseudomonas aeruginosa* using microarray technique. Front. Microbiol..

[65] Huang T.P., Wong A.C. (2007). Extracellular fatty acids facilitate flagella-independent translocation by *Stenotrophomonas maltophilia*. Res. Microbiol..

[66] Ryan R.P., Fouhy Y., Garcia B.F., Watt S.A., Niehaus K., Yang L., Tolker-Nielsen T., Dow J.M. (2008). Interspecies signalling via the *Stenotrophomonas maltophilia* diffusible signal factor influences biofilm formation and polymyxin tolerance in *Pseudomonas aeruginosa*. Mol. Microbiol..

[67] Inoue T., Shingaki R., Fukui K. (2008). Inhibition of swarming motility of *Pseudomonas aeruginosa* by branched-chain fatty acids. FEMS Microbiol. Lett..

[68] Boon C., Deng Y., Wang L.-H., He Y., Xu J.-L., Fan Y., Pan S.Q., Zhang L.-H. (2008). A novel DSF-like signal from *Burkholderia cenocepacia* interferes with *Candida albicans* morphological transition. ISME J..

[69] Deng Y., Boon C., Chen S., Lim A., Zhang L.-H. (2013). *Cis*-2-dodecenoic acid signal modulates virulence of *Pseudomonas aeruginosa* through interference with quorum sensing systems and T3SS. BMC Microbiol..

[70] Amari D.T., Marques C.N.H., Davies D.G. (2013). The putative enoyl-coenzyme A hydratase DspI is required for production of the *Pseudomonas aeruginosa* biofilm dispersion autoinducer *cis*-2-decenoic acid. J. Bacteriol..

[71] Bi H., Christensen Q.H., Feng Y., Wang H., Cronan J.E. (2012). The *Burkholderia cenocepacia* BDSF quorum sensing fatty acid is synthesized by a bifunctional crotonase homologue having both dehydratase and thioesterase activities. Mol. Microbiol..

[72] Ryan R.P., Dow J.M. (2011). Communication with a growing family: Diffusible signal factor (DSF) signaling in bacteria. Trends Microbiol..

[73] Deng Y., Schmid N., Wang C., Wang J., Pessi G., Wu D., Lee J., Aguilar C., Ahrens C.H., Chang C. (2012). *Cis*-2-dodecenoic acid receptor RpfR links quorum-sensing signal perception with regulation of virulence through cyclic dimeric guanosine monophosphate turnover. Proc. Natl. Acad. Sci. USA..

[74] Huedo P., Yero D., Martínez-Servat S., Estibariz I., Planell R., Martínez P., Ruyra A., Roher N., Roca I., Vila J. (2014). Two different *rpf* clusters distributed among a population of *Stenotrophomonas maltophilia* clinical strains display differential diffusible signal factor production and virulence regulation. J. Bacteriol..

[75] Marques C.N.H., Morozov A., Planzos P., Zelaya H.M. (2014). The fatty acid signaling molecule *cis*-2-decenoic acid increases metabolic activity and reverts persister cells to an antimicrobial-susceptible state. Appl. Environ. Microbiol..

[76] Gutierrez M., Choi M.H., Tian B., Xu J., Rho J.K., Kim M.O., Cho Y.-H., Yoon S.C. (2013). Simultaneous inhibition of rhamnolipid and polyhydroxyalkanoic acid synthesis and biofilm formation in *Pseudomonas aeruginosa* by 2-bromoalkanoic acids: Effect of inhibitor alkyl-chain-length. PLoS ONE.

[77] Su Z., Peng L., Worthington R.J., Melander C. (2011). Evaluation of 4,5-disubstituted-2-aminoimidazole-triazole conjugates for antibiofilm/antibiotic resensitization activity against MRSA and *Acinetobacter baumannii*. ChemMedChem.

[78] Ren D., Zuo R., Barrios A.F.G., Bedzyk L.A., Eldridge G.R., Pasmore M.E., Wood T.K. (2005). Differential gene expression for investigation of *Escherichia coli* biofilm inhibition by plant extract ursolic acid. Appl. Environ. Microbiol..

[79] Qin N., Tan X., Jiao Y., Liu L., Zhao W., Yang S., Jia A. (2014). RNA-Seq-based transcriptome analysis of methicillin-resistant *Staphylococcus aureus* biofilm inhibition by ursolic acid and resveratrol. Sci. Rep..

[80] Garo E., Eldridge G.R., Goering M.G., Pulcini E.D., Hamilton M.A., Costerton J.W., James G.A. (2007). Asiatic acid and corosolic acid enhance the susceptibility of *Pseudomonas aeruginosa* biofilms to tobramycin. Antimicrob. Agents Chemother..

[81] Potterat O., Hostettmann K., Höltzel A., Jung G., Diehl P.A., Petrini O. (1997). Boophiline, an antimicrobial sterol amide from the cattle tick *Boophilus microplus*. Helv. Chim. Acta.

[82] Zimmer K.R., Macedo A.J., Giordani R.B., Conceição J.M., Nicastro G.G., Boechat A.L., Baldini R.L., Abraham W.-R., Termignoni C. (2013). A steroidal molecule present in the egg wax of the tick *Rhipicephalus* (*Boophilus*) *microplus* inhibits bacterial biofilms. Environ. Microbiol..

[83] Forenza S., Minale L., Riccio R., Fattorusso E. (1971). New bromo-pyrrole derivatives from the sponge *Agelas oroides*. J. Chem. Soc. D.

[84] Kelly S.R., Jensen P.R., Henkel T.P., Fenical W., Pawlik J.R. (2003). Effects of Caribbean sponge extracts on bacterial attachment. Aquat. Microb. Ecol..

[85] Žula A., Kikelj D., Ilaš J. (2013). 2-Aminoimidazoles in medicinal chemistry. Mini Rev. Med. Chem..

[86] Richards J.J., Reyes S., Stowe S.D., Tucker A.T., Ballard T.E., Mathies L.D., Cavanagh J., Melander C. (2009). Amide isosteres of oroidin: Assessment of antibiofilm activity and *C. elegans* toxicity. J. Med. Chem..

[87] Ballard T.E., Richards J.J., Aquino A., Reed C.S., Melander C. (2009). Antibiofilm activity of a diverse oroidin library generated through reductive acylation. J. Org. Chem..

[88] Hodnik Ž., Łoś J.M., Žula A., Zidar N., Jakopin Ž., Łoś M., Dolenc M.S., Ilaš J., Węgrzyn G., Mašič L.P. (2014). Inhibition of biofilm formation by conformationally constrained indole-based analogues of the marine alkaloid oroidin. Bioorganic Med. Chem. Lett..

[89] Wright C.J., Wu H., Melander R.J., Melander C., Lamont R.J. (2014). Disruption of heterotypic community development by *Porphyromonas gingivalis* with small molecule inhibitors. Mol. Oral Microbiol..

[90] Polo A., Foladori P., Ponti B., Bettinetti R., Gambino M., Villa F., Cappitelli F. (2014). Evaluation of zosteric acid for mitigating biofilm formation of *Pseudomonas putida* isolated from a membrane bioreactor system. Int. J. Mol. Sci..

[91] Villa F., Pitts B., Stewart P.S., Giussani B., Roncoroni S., Albanese D., Giordano C., Tunesi M., Cappitelli F. (2011). Efficacy of zosteric acid sodium salt on the yeast biofilm model *Candida albicans*. Microb. Ecol..

[92] Villa F., Remelli W., Forlani F., Vitali A., Cappitelli F. (2012). Altered expression level of *Escherichia coli* proteins in response to treatment with the antifouling agent zosteric acid sodium salt. Environ. Microbiol..

[93] Cattò C., Dell’Orto S., Villa F., Villa S., Gelain A., Vitali A., Marzano V., Baroni S., Forlani F., Cappitelli F. (2015). Unravelling the structural and molecular basis responsible for the anti-biofilm activity of zosteric acid. PLoS ONE.

[94] Li W., Estrada-de los Santos P., Matthijs S., Xie G.L., Busson R., Cornelis P., Rozenski J., de Mot R. (2011). Promysalin, a salicylate-containing *Pseudomonas putida* antibiotic, promotes surface colonization and selectively targets other *Pseudomonas*. Chem. Biol..

[95] Steele A.D., Knouse K.W., Keohane C.E., Wuest W.M. (2015). Total synthesis and biological investigation of (−)-promysalin. J. Am. Chem. Soc..

[96] Clatworthy A.E., Pierson E., Hung D.T. (2007). Targeting virulence: A new paradigm for antimicrobial therapy. Nat. Chem. Biol..

[97] Romilly C., Lays C., Tomasini A., Caldelari I., Benito Y., Hammann P., Geissmann T., Boisset S., Romby P., Vandenesch F. (2014). A non-coding RNA promotes bacterial persistence and decreases virulence by regulating a regulator in *Staphylococcus aureus*. PLoS Pathog..

[98] Rasmussen T.B., Skindersoe M.E., Bjarnsholt T., Phipps R.K., Christensen K.B., Jensen P.O., Andersen J.B., Koch B., Larsen T.O., Hentzer M. (2005). Identity and effects of quorum-sensing inhibitors produced by *Penicillium.* species. Microbiology.

[99] Sepehr S., Rahmani-Badi A., Babaie-Naiej H., Soudi M.R. (2014). Unsaturated fatty acid, *cis*-2-decenoic acid, in combination with disinfectants or antibiotics removes pre-established biofilms formed by food-related bacteria. PLoS ONE.

[100] Deng Y., Lim A., Lee J., Chen S., An S., Dong Y.-H., Zhang L.-H. (2014). Diffusible signal factor (DSF) quorum sensing signal and structurally related molecules enhance the antimicrobial efficacy of antibiotics against some bacterial pathogens. BMC Microbiol..

[101] Rahmani-Badi A., Sepehr S., Mohammadi P., Soudi M.R., Babaie-Naiej H., Fallahi H. (2014). A combination of *cis*-2-decenoic acid and antibiotics eradicates pre-established catheter-associated biofilms. J. Med. Microbiol..

[102] García-Contreras R., Maeda T., Wood T.K. (2015). Can resistance against quorum-sensing interference be selected?. ISME J..

[103] Johns B.E., Purdy K.J., Tucker N.P., Maddocks S.E. (2015). Phenotypic and genotypic characteristics of small colony variants and their role in chronic infection. Microbiol. Insights.

[104] García-Contreras R., Peréz-Eretza B., Jasso-Chávez R., Lira-Silva E., Roldán-Sánchez J.A., González-Valdez A., Soberón-Chávez G., Coria-Jiménez R., Martínez-Vázquez M., Alcaraz L.D. (2015). High variability in quorum quenching and growth inhibition by furanone C-30 in *Pseudomonas aeruginosa* clinical isolates from cystic fibrosis patients. Pathog. Dis..

[105] Grandclément C., Tannières M., Moréra S., Dessaux Y., Faure D.D. (2015). Quorum quenching: Role in nature and applied developments. FEMS Microbiol. Rev..

[106] Gopal R., Lee J.H., Kim Y.G., Kim M.S., Seo C.H., Park Y. (2013). Anti-microbial, anti-biofilm activities and cell selectivity of the NRC-16 peptide derived from witch flounder, *Glyptocephalus cynoglossus*. Mar. Drugs.

[107] Jiang F., Deng Y., Yeh C.K., Sun Y. (2014). Quaternized chitosans bind onto preexisting biofilms and eradicate pre-attached microorganisms. J. Mater. Chem. B Mater. Biol. Med..

[108] Zupancic S., Kocbek P., Baumgartner S., Kristl J. (2015). Contribution of nanotechnology to improved treatment of periodontal disease. Curr. Pharm. Des..

[109] Rawson M., Haggard W., Jennings J.A. (2014). Osteocompatibility of biofilm inhibitors. Open Orthop. J..

